# Effects of Dietary Yeast, *Saccharomyces cerevisiae*, and Costmary, *Tanacetum balsamita*, Essential Oil on Growth Performance, Digestive Enzymes, Biochemical Parameters, and Disease Resistance in Nile Tilapia, *Oreochromis niloticus*

**DOI:** 10.1155/2024/1388002

**Published:** 2024-08-27

**Authors:** Hossein Adineh, Morteza Yousefi, Basim S. A. Al Sulivany, Ehsan Ahmadifar, Mohammad Farhangi, Seyyed Morteza Hoseini

**Affiliations:** ^1^ Department of Fisheries Faculty of Agriculture and Natural Resources Gonbad Kavous University, Gonbad Kavous, Golestan, Iran; ^2^ Department of Veterinary Medicine RUDN University, 6 Miklukho-Maklaya Street, Moscow 117198, Russia; ^3^ Department of Biology College of Science University of Zakho, Duhok, Zakho, Iraq; ^4^ Department of Fisheries Faculty of Natural Resources University of Zabol, Zabol, Iran; ^5^ Inland Waters Aquatics Resources Research Center Iranian Fisheries Sciences Research Institute Agricultural Research Education and Extension Organization, Gorgan, Iran

## Abstract

The present study assessed the effects of dietary yeast, *Saccharomyces cerevisiae*, and costmary, *Tanacetum balsamita*, essential oil on growth performance, biochemical parameters, and disease resistance of Nile tilapia, *Oreochromis niloticus*. Four diets containing 1 g/kg yeast (Sc), 0.1 g/kg costmary essential oil (Tb), 1 g/kg yeast + 0.1 g/kg costmary essential oil (Sc + Tb), and without Sc and Tb (control) were formulated and fed (2.5% per day) to triplicate groups of fish (average: 9.8 g; SD : 0.12) for 8 weeks. Each replicate was a 70-L tank, stocked with 25 fish. Then, the fish were intraperitoneally challenged by *Aeromonas hydrophila*, and blood samples were taken from the fish before and 12 hr postinfection. All experimental groups showed significantly higher growth performance and feed efficiency, compared to the control, and the highest values were related to Sc + Tb treatment (*P*  < 0.001). Sc group showed significant elevations in the intestinal amylase, lipase, and protease activities, but Tb group showed only elevation in lipase activity. The highest amylase (*P*=0.026), lipase (*P*=0.036), and protease (*P*=0.009) activities were observed in Sc + Tb treatment. The postchallenge survival of Sc (70.0%), Tb (73.3%), and Sc + Tb (76.6%) treatments were significantly (*P*=0.038) higher than the control (56.6%). Bacterial challenge significantly increased plasma cortisol, glucose, malondialdehyde, glutathione peroxidase, and superoxide dismutase, but decreased lysozyme, alternative complement, albumin, globulin, and catalase (*P*  < 0.001). The Tb treatment showed improvements in plasma antioxidant, immunological, and biochemical parameters, compared to the Sc treatment. The Sc + Tb treatment showed the highest albumin, globulin, lysozyme, total immunoglobulin, alternative complement, glutathione peroxidase, and superoxide dismutase, but the lowest cortisol, glucose, malondialdehyde, and catalase, before/after the challenge (*P*  < 0.001). In conclusion, dietary Sc + Tb supplementation positively affects growth performance, antioxidant, and immunological responses, thereby augments resistance of Nile tilapia to *A. hydrophila* infection.

## 1. Introduction

Aquaculture is a rapidly expanding sector in the food industry, with the Food and Agricultural Organization (FAO) reporting an increase in the consumption of aquaculture products from around 16% in 1990 to around 50% in 2020 [1]. One of the key fish in freshwater aquaculture is Nile tilapia, *Oreochromis niloticus* (L.), and its global production surged from 380,000 tons in 1990 to 6 million tons in 2018 [2]. However, disease loss is an obstacle in the progression of Nile tilapia aquaculture. Bacterial infections are one of the most common causes of fish illness, with *Aeromonas hydrophila* is a common widespread pathogen that infests a wide variety of aquaculture species [3]. It is well established that *A. hydrophila* infection is a common disease of farmed tilapia, worldwide [4, 5]. Due to the negative effects of antibiotics on environment and raise of the resistant bacteria, the world authorities determined tight regulations for antibiotic application [6, 7]; hence, aquaculturists seek for the methods of boosting fish health to prevent/mitigate damages from infections with opportunistic bacteria.

An early response of fish to combat bacterial invasion is activation of the innate immune system, particularly circulating lysozyme and alternative complement activities [8]. These two agents have remarkable roles in killing bacteria, although the latter also contribute in opsonization and inflammation [9, 10]. Studies have shown that *A. hydrophila* induces stress in fish, characterized by increase in cortisol levels in circulation [11, 12], which is detrimental for the proper immune responses [13]. Furthermore, bacterial infection activates the antioxidant system and induces oxidative stress [14, 15]. Oxidative stress can depress innate immune responses to bacterial infection, as some responses such as phagocytosis and respiratory burst activity produces oxidative compounds to kill the pathogens [16]. As the fish body can handle certain levels of oxidative compounds, occurrence of oxidative stress limits the oxidative functions of the innate immune system. Thus, strengthening the immune responses and antioxidant capacity, along with mitigating stress can improve disease resistance in fish.

A large body of evidence demonstrated that dietary additives can improve resistance against disease in fish through improving immune power, antioxidant capacity, and mitigating stress [17, 18, 19]. Probiotics are well-known immunostimulants in aquaculture that have other benefits in the host including surging the innate immune power, antioxidant capacity, and disease resistance [20, 21]. Among them, yeast, *Saccharomyces cerevisiae*, is a famous probiotic due to the presence of beta-glucans and mannan oligosaccharides in its membrane and high concentrations of nucleotides [22, 23]. Studies on Nile tilapia have shown that yeast is an efficient feed additive because it can improve growth and feed efficiency [24, 25, 26, 27, 28], stress resistance [27], morphological structure of the liver and intestine [25, 26, 28], and immune responses and resistance against *A. hydrophila* infection [24, 25, 26, 27]. Herbal materials are extensively used as feed additives in aquaculture, thanks to their health benefits such as improving antioxidant capacity, immune responses, and stress and disease resistance (reviewed by Abarike et al. [29] and Elumalai et al. [30]). Nowadays, benefits of numerous herbal additives are investigated in Nile tilapia, emphasizing on their growth promoting, antioxidant, and immune-boosting effects (reviewed by Gabriel [31]); however, there are other herbal materials not examined in this species. Costmary, *Tanacetum balsamita*, is a fragrant plant that can be found in the northern hemisphere [32] and recognized as safe by U.S. Food and Drug Administration and the Environmental Protection Agency [33]. Carvone, a compound with antioxidant, antimicrobial, and immunostimulant properties [34, 35], is abundant in the essential oil derived [36]. Costmary essential oil has been recently used in aquaculture nutrition. Feeding common carp, *Cyprinus carpio*, with diets containing costmary essential oil resulted in higher growth performance, antioxidant status, and immune responses, as well as lower stress [37]. However, there are no data about its benefits in other fish, including Nile tilapia, raising the need for further studies.

Considering the lack of data about the roles of costmary essential oil in Nile tilapia, the aim of the present study was to illustrate this topic for the first time. Bearing in mind that dietary yeast has several health benefits and growth-promoting effects in this species, the combined effects of dietary yeast and costmary essential oil were also assessed in the present study.

## 2. Materials and Methods

### 2.1. Experimental Diets

In this experiment, a control diet and three different diets were prepared (Table 1). Costmary essential oil was purchased from Giah Essence Pharmaceutical Company (Gorgan, Iran). *S. cerevisiae* (IBRC-M 30004) was obtained from the National Center for Genetic and Biological Resources of Iran. The experimental diets contained yeast at a concentration of 1 g/kg, costmary essential oil at a concentration of 100 mg/kg, or a combination of yeast and costmary at concentrations of 1 g/kg and 100 mg/kg, respectively. The feedstuffs were mixed together and then the yeast and costmary were added to the mixture after combining with the dietary oils. To moisten the mixture, 400 mL/kg of water were added and the resulting dough was formed into feed pellets by passing it through a mesh. After drying the pellets overnight, crude protein, crude fat, crude ash, and dry matter of the diets were measured according to standard methods [38].

### 2.2. Fish Rearing Conditions

Four hundred Nile tilapia juveniles weighing approximately 9.7–9.8 g were obtained from a local farm and acclimatized to the laboratory conditions for 2 weeks. During the acclimation, the fish were kept in a 1.5 m^3^ tank and fed the control diet. After acclimation, 300 fish with similar weights and no external abnormalities were randomly distributed into 12 tanks (70 L), with 25 fish per tank. All the tanks were equipped with an air–stone for aeration and 30% of the water was daily replaced by clean water. The abovementioned diets were assigned to a batch of three tanks an offered to the fish based on 2.5% of biomass for 8 weeks. The feed amounts were adjusted biweekly, after recording biomass of the tanks. Water quality was recorded over the feeding period using electrochemical probes (Hach Co., Orlando, USA) and photometer (Palintest Co., Birmingham, UK), being as follows: temperature: 26.91 ± 0.37°C, dissolved oxygen: 6.50 ± 0.36 mg/L, pH: 7.58 ± 0.20, total alkalinity: 402.10 ± 10.57 mg CaCO_3_/L, and unionized ammonia: 0.060 ± 0.013 mg/L.

At the end of the rearing period, measurements were taken for final weight (FW), weight gain (WG), specific growth rate (SGR), and feed conversion ratio (FCR) using the following formulas:

(1) $$\begin{array}{c} \text{WG}\left(\text{\%}\right)=100\times \frac{\text{Final weight}-\text{initial weight}}{\text{Initial weight}}, \end{array}$$

(2) $$\begin{array}{c} \text{FCR}=\frac{\text{Feed intake}}{\text{Final weight}-\text{initial weight}}, \end{array}$$

(3) $$\begin{array}{c} \text{SGR}\left(\%/\text{day}\right)=100\times \frac{\text{Ln}\left(\text{final weight}\right)\text{-ln}\left(\text{initial weight}\right)}{\text{Rearing period}\left(\text{days}\right)}. \end{array}$$

### 2.3. Intestinal Sampling and Digestive Enzymes Analysis

At the end of the experiment, the activity of digestive enzymes was determined in the fish intestine. Fish (three per tank) were caught by a dip net, killed by a sharp blow on the head, and dissected by scissors. The whole intestine was carefully cut, placed in liquid nitrogen, and transferred to −70°C freezer. The samples were crushed in a mortar on ice for 3 min; then two volumes (v/w) of a phosphate buffer (pH 7.0) were added to the homogenate and mixed well. The mixture was centrifuged at 4°C (13,000 *g*; 20 min), and the resulting top liquid was used for analysis. A solution of 0.3% starch was used as substrate to measure amylase activity as per Langlois et al.'s [39] study. Lipase activity in the intestine samples was determined based on the hydrolysis of p-nitrophenyl myristate [40]. Total protease activity in the intestine was determined using 1% casein as substrate, based on the AZO-casein method [41]. Soluble protein amount of the samples was determined according to Bradford [42] and the enzymes' activities were expressed based on the soluble protein concentration (specific activity).

### 2.4. Bacterial Challenge

After the 8-week rearing, all fish were challenged by *A. hydrophila*. The bacterium (ATCC 7966) was obtained from the Iranian Biological Resource Center (Tehran, Iran) in lyophilized form. It was first dissolved in 5 mL of sterile water and spread on plates containing on nutrient agar for 48 hr. Then, a suspension of 1 × 10^8^ cells/mL was prepared in 8.5 g/L sterile NaCl. Thirteen fish from each tank were anesthetized using a clove extract solution (3 g/L) and intraperitoneally injected with 100 *µ*L of the bacterial suspension [43]. Also, 10 fish from the control tanks were randomly selected and injected with 100 *µ*L of NaCl solution to serve as a negative control group. The fish mortality was monitored for 14 days in their corresponding tanks.

### 2.5. Blood Sampling and Plasma Analysis

Before (basal) and 12 hr after the bacterial challenge, three fish were randomly netted and anesthetized in a clove extract solution (3 g/L). Heparinized syringes were used to collect blood samples from the fish caudal vein. After the sampling, plasma was separated from the whole blood by centrifuging the samples at 4°C (5,000 *g*; 10 min). The plasma samples were kept at −70°C until analysis.

Plasma cortisol levels were determined by ELISA method using a commercial kit (IBL, Gesellschaft für Immunchemieund Immunbiologie, Germany) following Hoseini et al.'s [43] study. The kit was designed based on competitive ELISA method. Cortisol antigen was coated on the internal surface of a 96-well plate, which reacted with the sample cortisol. Inter- and intra-assay coefficients of variation were 8.69% and 10.2%, respectively. Glucose, total protein, and albumin levels were measured using colorimetric kits provided by Zist Chem Co. (Tehran, Iran). Plasma globulin levels were determined by subtracting the levels of plasma total protein and albumin.

Commercial kits (ZellBio GmbH, Veltinerweg, Germany) were used to measure antioxidant parameters in a microplate reader. Plasma malondialdehyde (MDA) concentration was determined based on the amount of thiobarbituric reactive substances at 95°C for 1 hr [44]. The activity of plasma superoxide dismutase (SOD) was determined based on Cytochrome C reduction rate as suggested by Hoseini et al. [44]. Glutathione peroxidase (GPx) activity was determined based on the conversion of reduced glutathione to glutathione disulfide and reaction of the remaining reduced glutathione with 5,5′-dithiobis-(2-nitrobenzoic acid) [44]. Plasma catalase (CAT) activity was determined based on the decomposition of hydrogen peroxide and reaction of the remaining hydrogen peroxide with ammonium molybdate [45].

Total immunoglobulin (Ig) levels in the plasma samples were measured by mixing equal volumes of the samples and polyethylene glycol (PEG) 12%. After 2 hr shaking at room temperature, the sample Ig was separated by centrifugation (15 min, 5,000 rpm). Total protein levels of the original sample and PEG-treated one were subtracted to calculate total Ig [46]. Alternative complement (ACH50) activity in the plasma samples was determined according to Yano [47]. Serial dilutions of the plasma samples were prepared using gelatin–barbital buffer containing magnesium and EGTA. The diluted samples were mixed with diluted sheep erythrocyte and incubated for 90 min. The plasma volume giving 50% hemolysis was used for ACH50 calculation. In order to measure the lysozyme activity, the plasma samples were mixed with a suspension of *Micrococcus luteus* and decrease in the optical density of the mixture was recorded for 5 min. Each 0.001 decrease in optical density per minute was considered as one unit of lysozyme activity, as described by Ellis [48].

### 2.6. Statistical Analysis

The software Statistical Package for the Social Sciences (SPSS) v.22 was operated for statistical analysis of the data. The data were first checked for normal distribution and homoscedasticity using the Shapiro–Wilk's and Levene's tests, respectively. Data of the fish growth performance and intestinal digestive enzymes activities were analyzed by one-way ANOVA, whereas plasma cortisol, glucose, SOD, CAT, GPx, MDA, lysozyme, total Ig, and ACH50 were analyzed by two-way ANOVA (dietary × sampling times). Pair comparisons among the treatments were performed by the Duncan test. Postchallenge survival data were analyzed by Kruskal–Wallis and Mann–Whitney *U* tests, as they did not meet ANOVA assumptions. *α* was set to 0.05 in all analyses and the data were presented as mean ± standard deviation.

## 3. Results

Growth performance, feed efficiency, and survival of the fish are shown in Table 2. Dietary supplementation with Sc, Tb, and Sc + Tb significantly improved growth performance and feed efficiency, compared to control. The highest growth performance and feed efficiency values were related to Sc + Tb treatment. There were no mortalities among the treatments.

Intestinal activities of the digestive enzymes are presented in Table 3. Intestinal lipase activity significantly increased in the experimental groups, compared to the control. The Sc and Sc + Tb treatments exhibited significantly higher intestinal amylase and protease activity, compared to the control. No significant differences were observed in the intestinal trypsin and chymotrypsin among the treatments.

**Figure 1:**
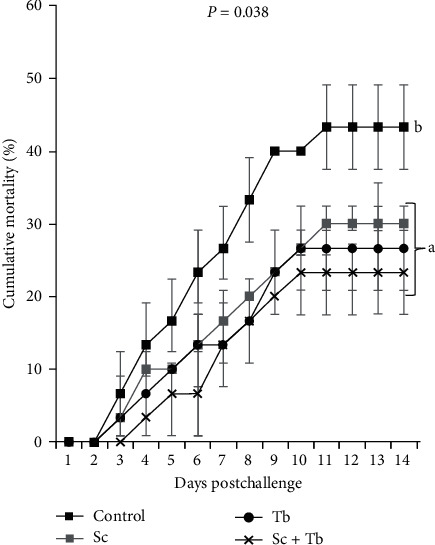
Cumulative mortality percentages of Nile tilapia fed diets containing *S. cerevisiae* (Sc), costmary essential oil (Tb), and *S. cerevisiae* + costmary essential oil (Sc + Tb) and challenged with *A. hydrophila*. Different letters show significant differences among the treatments (*n* = 3; Mann–Whitney U).

After the bacterial challenge, the experimental groups exhibited significantly lower mortality than the control. The fish mortalities were 43.3%, 30%, 26.7%, and 23.3% in the control, Sc, Tb, and Sc + Tb treatments, respectively (Figure 1).

There were interaction effects of diet and bacterial challenge on the plasma albumin and globulin levels (Table 4). Before the challenge, Tb and Sc + Tb treatments exhibited significantly higher albumin concentrations than the control. After the challenge, plasma albumin concentration significantly decreased in Tb treatment, but it was comparable to the control and Sc treatments. The highest plasma albumin level was related to Sc + Tb treatment, after the challenge. Plasma globulin of Sc + Tb treatment was significant higher than the control, before the fish was challenged. The bacterial challenge significantly decreased plasma globulin levels in treatments, except Tb treatment; nevertheless, the experimental groups showed similar globulin levels and significantly higher than the control.

Diet and bacterial challenges significantly affected plasma cortisol and glucose levels. The experimental groups showed similar glucose levels and significantly lower than the control. Plasma cortisol levels of Tb and Sc + Tb were significantly lower than the control. The bacterial challenge significantly increased plasma cortisol and glucose (Table 4).

Diet and bacterial challenge significantly affected plasma lysozyme activity. The experimental groups had similar lysozyme activities and significantly higher than control and bacterial challenge significantly decreased plasma lysozyme activity (Table 5). Total Ig levels of plasma significantly increased in the experimental groups, compared to control. Plasma total Ig of Tb group was significantly higher than Sc group and the highest level was observed in Sc + Tb group (Table 5). There was an interaction effect of diet and bacterial challenge on plasma ACH50 activities. Before the challenge, Tb and Sc + Tb treatment showed significantly higher ACH50 compared to the control. The bacterial challenge significantly decreased ACH50 activity but the experimental groups showed significantly higher ACH50 than control. Plasma ACH50 activities in the Tb and Sc + Tb treatment were significantly higher than that of the Sc treatment, after the bacterial challenge.

Diet and bacterial challenge significantly affected plasma MDA levels. The experimental groups had similar MDA levels and significantly lower than control and bacterial challenge significantly increased plasma MDA activity (Table 6). There were interaction effects of diet and bacterial challenge on plasma GPx, SOD, and Cat activities. Before the challenge, plasma GPx activities of Tb and Sc + Tb were significantly higher than the control, but after the challenge all experimental groups had significantly higher plasma GPx activities, compared to the control. All treatments showed significant decrease in plasma GPx activity, but Sc + Tb had significantly higher activity than the other treatments. Before the challenge, all experimental treatments had significantly higher plasma SOD activities than control. Bacterial challenge significantly increased SOD activity in the experimental groups, but not control. The highest SOD activity after the challenge was related to Sc + Tb treatment. Before the challenge, Tb and Sc + Tb treatments showed significantly lower CAT activities than the control. Bacterial challenge significantly decreased CAT activities in the experimental groups, but not control.

## 4. Discussion

The present study showed that dietary yeast and costmary essential oil are capable of improving growth performance and feed efficiency, but they synergically improve these parameters. The results are in line with previous studies that reported an improvement in growth performance and feed efficiency in Nile tilapia fed diet supplemented with 1 g/kg [25, 26] or higher levels of yeast [4, 27]. These effects may be associated with the yeast cell wall, since inactivated yeast [26] and yeast cell wall components [49] at 1 g/kg improved growth and feed efficiency in Nile tilapia. The yeast cell wall contains beta-glucans and mannan oligosaccharides, which found to increase intestinal digestive enzymes [50, 51, 52]. Thus, increased intestinal digestive enzymes in Sc treatment may explain the higher growth rate observed in this study. Similarly, growth promotion by dietary costmary essential oil may be due to the same reasons, which is supported by the results obtained in common carp-fed diets supplemented by costmary essential oil [37]. Interestingly, this study showed that dietary yeast and costmary essential oil have synergistic effects on growth performance and feed efficiency of Nile tilapia independent of digestive enzymes. Whether this combination facilitates nutrient absorption and/or energy allocation needs further assays.

In this study, all the experimental treatments showed better resistance against *A. hydrophila* infection; although nominally, the fish resistance was in the following order: Sc < Tb < Sc + Tb. Even though it is well-established that dietary yeast can increase resistance against *A. hydrophila* infection in Nile tilapia [24, 25, 26, 27], there is no data regarding the efficacy of costmary essential oil in fish. Several factors may have contributed to the improved disease resistance in Tb and Sc + Tb treatments, which are discussed below.

One of the benefits of using additives in aquafeeds is stress mitigation [53]. This is an important feature of the additives, keeping in mind the stressful nature of fish rearing in captivity and drawbacks of stress on the immune system and disease resistance. The present study showed that plasma cortisol and glucose levels decreased in the experimental groups, compared to the control. In support of these findings, costmary essential oil showed similar effects on plasma stress markers of common carp, either under normal or stressful (ammonia exposure) conditions [37]. Beta-glucan in the yeast cell wall has been shown to reduce cortisol and glucose in fish [54], which may explain the present findings. Studies have shown that stress mitigation by feed additives is a determining factor postchallenge survival, probably due to the allocation of available energy to fighting against pathogens. For example, dietary administration of beta-glucan + mannan oligosaccharide to rainbow trout, *Oncorhynchus mykiss* [11], microbial levan to ruho, *Labeo rohita* [12], and beta-glucan to pacu, *Piaractus mesopotamicus* [54] significantly decreased stress and mortality associated with *A. hydrophila* infection, which supports the present results.

Albumin and globulin are synthetized in the fish liver and decreases in their concentrations can be indicators of hepatic issues [55]. Accordingly, Tb and Sc + Tb treatments may have healthier livers than the control treatment, since they had higher plasma albumin and globulin levels. Supporting these findings, previous studies on Nile tilapia showed elevations in blood albumin and globulin levels after dietary yeast (or yeast cell wall/extract) supplementation [28, 49, 56] along with healthier livers [28]. Furthermore, costmary essential oil supplementation showed an increase in the blood albumin and decrease in the blood alanine aminotransferase and aspartate aminotransferase in common carp, which may be due to a healthier liver [37]. During disease, the fish body allocates more energy to combat pathogens, thus, synthesis of new proteins may collapse [57]. Infectious bacteria, like *A. hydrophila* [49, 58], damage the fish liver tissue that may further decrease hepatic protein synthesis. This partially explains the decrease in plasma albumin/globulin levels after the challenge in the present study. Supporting this theory, previous studies showed that increase in plasma albumin/globulin levels by dietary approaches decreased fish mortality after a bacterial challenge [49, 56, 58, 59].

It has been shown that lysozyme activity increases after bacterial infections in fish, emphasizing its role in pathogen elimination (reviewed by Saurabh and Sahoo [60]). However, some pathogens can deactivate lysozyme by production of lysozyme inhibitors. *A. hydrophila* produces periplasmic proteins [61] that inactivate lysozyme [62, 63], which explains the fall in the plasma lysozyme activity after challenge in the present study. On the other hand, both yeast [64, 65] and costmary essential oil [37] have been reported to augment plasma lysozyme activity under either normal or stressful (ammonia exposure or bacterial challenge) conditions, which support the present findings.

Complement proteins are one of the humoral immune components, involving in opsonization, cell lysis, and inflammatory responses [10]. The concentration of complement proteins increases in response to pathogen invasions, such as *A. hydrophila* [66, 67]. Nevertheless, the present results showed that complement activity significantly decreased after the challenge, which is due to the fact that *A. hydrophila* can degrade the C3 component of the complement system by the action of certain metaloproteins [68]. Studies have reported that dietary yeast [64, 69] or costmary essential oil [37] increase complement activity, which support the present results. Thus, it is speculated that the higher postchallenge survival in the present study can be due to the higher prechallenge elevation in the plasma lysozyme and complement activities that probably improved *A. hydrophila* elimination in the fish body.

Although Ig are components of the adaptive immunity [70], it has been shown that basal levels of circulating Ig are sensitive to dietary immunostimulant, and higher basal Ig has been observed in fish with higher survival after bacterial challenges [71, 72, 73]. The present results are in line with previous studies reporting elevations in fish circulating Ig levels after dietary administration of yeast products [74, 75] or costmary essential oil [37].

The antioxidant system importantly contributes to immune responses, as enough antioxidant power is needed to proper action of immune factors with oxidative functions (e.g., phagocytosis, superoxide anion production, and nitric oxide production) [16]. The decrease in lipid peroxidation in the experimental groups seems to be a result of the elevations in SOD and GPx activities, as they are responsible for detoxifying superoxide ions and hydroxides [76]. Although CAT is responsible for hydrogen peroxide detoxification, it acts at high substrate concentrations and GPx is the main detoxifier of lower concentrations of hydrogen peroxide [77, 78]. Hence, the present results suggest that the experimental groups had better antioxidant capacity than control. Yeast can stimulate antioxidant system by the presence of beta-glucans and mannan oligosaccharide in its cell wall, as observed in previous studies [74, 79, 80]. Improved antioxidant power by dietary yeast or its products has improved nitric oxide production [80] respiratory burst activity [75, 79], or myeloperoxidase [65], thereby improved disease resistance. On the other hand, costmary essential oil contains high levels of carvone, a compound known as antioxidant [81], which justifies the present findings.

The present study showed that dietary 1 g/kg yeast and 100 mg/kg costmary essential oil can similarly improve growth performance and feed efficiency in Nile tilapia, but the highest responses are obtained if these supplements are simultaneously used. Focusing on the biochemical parameters, both additives successfully improve antioxidant and immune responses and decrease stress; however, costmary essential oil has higher benefits than yeast and the best situation is when both feed additives are used together. According to the present results, higher disease resistance in the fish yeast and costmary essential oil is due to healthier liver and protein production, lower stress and energy expenditure, and higher antioxidant capacity and immune responses.

## Figures and Tables

**Table 1:** Ingredients and chemical composition of the basal diet (g/kg diet on dry matter basis).

Ingredients (g/kg diet)	Experimental diets			
	Control	Sc	Tb	Sc + Tb
Fishmeal^1^	150	150	150	150
Soybean meal	315	315	315	315
Wheat meal	378	378	378	378
Fish oil	7	7	7	7
Soybean oil	7	7	7	7
Corn flour	120	120	120	120
Gluten	4	4	4	4
L-Lysine^2^	7	7	7	7
L-Methionine^2^	5	5	5	5
Vitamin premix^a^	2.5	2.5	2.5	2.5
Mineral premix^b^	2.5	2.5	2.5	2.5
Cellulose	2	1	1.9	0.9
Yeast	0	1	0	1
Costmary essential oil	0	0	0.1	0.1
Proximate chemical composition				
Dry matter	90.00	89.91	89.99	89.90
Crude protein (%)	30.87	30.85	30.86	30.85
Crude fat (%)	6.06	6.05	6.05	6.05
Crude ash (%)	5.66	5.66	5.66	5.66
Energy (kcal/kg)	3,943.26	3,939.34	3,942.86	3,938.94

^1^Pars-Kilka Corporation, Iran (nitrogen: 4.24%−4.36%, lipid: 8%–11%, ash: 11.6%, dry matter: 91%–93%); ^2^Morghe-Nojan Corporation, Tehran, Iran. ^a^Vitamin premix (per kg of diet): ascorbic acid (35%), 110 mg; riboflavin, 5 mg; thiamine, 5 mg; B_6_, 5 mg; B_12_, 0.025 mg; D_3_, 1,200 IU; vitamin A, 2,000 IU; vitamin E, 63 mg; K_3_, 2.5 mg; pantothenic acid, 20 mg; folic acid, 1.3 mg; inositol, 60 mg; niacin, 25 mg; and biotin, 0.05 mg; ^b^mineral premix (mg/kg of diet): magnesium, 2; manganese, 3.6; NaCl, 165; K, 50; iodine, 0.76; zinc, 8; iron, 38.7; copper, 5; and cobalt, 1.5.

**Table 2:** Growth performance of Nile tilapia fed with diets containing *S. cerevisiae* (Sc), costmary essential oil (Tb), and *S. cerevisiae* + costmary essential oil (Sc + Tb).

	Control	Sc	Tb	Sc + Tb	*P*-value
IW (g)	9.83 ± 0.058	9.78 ± 0.049	9.84 ± 0.053	9.82 ± 0.020	0.497
FW (g)	26.91 ± 0.24^a^	30.50 ± 0.48^b^	30.27 ± 0.17^b^	32.06 ± 0.12^c^	<0.001
WG (%)	173.78 ± 3.60^a^	211.60 ± 3.41^b^	207.40 ± 0.69^b^	226.27 ± 0.77^c^	<0.001
SGR (%/day)	1.79 ± 0.023^a^	2.02 ± 0.019^b^	2.00 ± 0.004^b^	2.11 ± 0.004^c^	<0.001
FCR	1.61 ± 0.027^c^	1.38 ± 0.032^b^	1.40 ± 0.014^b^	1.29 ± 0.004^a^	<0.001
Survival (%)	100	100	100	100	—

Different superscript letters within a row show significant differences (Duncan; *n* = 3). IW, initial weight; FW, final weight; WG, weight gain; SGR, specific growth rate; and FCR, feed conversion ratio.

**Table 3:** Intestinal activities of digestive enzymes of Nile tilapia fed with diets containing *S. cerevisiae* (Sc), costmary essential oil (Tb), and *S. cerevisiae* + costmary essential oil (Sc + Tb).

	Control	Sc	Tb	Sc + Tb	*P*-value
Amylase (U/mg Pr.)	38.30 ± 2.44^a^	44.00 ± 1.98^b^	43.05 ± 3.40^ab^	46.71 ± 2.43^b^	0.026
Lipase (U/mg Pr.)	1.48 ± 0.060^a^	1.69 ± 0.079^b^	1.62 ± 0.091^b^	1.63 ± 0.050^b^	0.036
Protease (U/mg Pr.)	3.72 ± 0.23^a^	4.54 ± 0.43^bc^	4.03 ± 0.14^ab^	4.87 ± 0.37^c^	0.009
Trypsin (*μ*mol/mg Pr.)	0.067 ± 0.006	0.077 ± 0.012	0.075 ± 0.005	0.085 ± 0.007	0.137
Chymotrypsin (*μ*mol/mg Pr.)	0.299 ± 0.017	0.324 ± 0.029	0.316 ± 0.014	0.352 ± 0.017	0.073

Different superscript letters within a row show significant differences (Duncan; *n* = 3).

**Table 4:** Plasma albumin, globulin, cortisol, and glucose levels of Nile tilapia fed with diets containing *S. cerevisiae* (Sc), costmary essential oil (Tb), and *S. cerevisiae* + costmary essential oil (Sc + Tb) and challenged with *A. hydrophila* (*n* = 3).

	Albumin (g/dL)	Globulin (g/dL)	Cortisol (ng/mL)	Glucose (mg/dL)
Before challenge				
Control	1.14 ± 0.02^ab^	1.21 ± 0.07^bc^	26.87 ± 1.95	79.57 ± 3.56
Sc	1.18 ± 0.02^bc^	1.33 ± 0.03^cd^	24.56 ± 1.28	69.28 ± 2.12
Tb	1.28 ± 0.01^e^	1.32 ± 0.09^cd^	22.52 ± 2.96	73.70 ± 2.15
Sc + Tb	1.25 ± 0.01^de^	1.39 ± 0.08^d^	21.67 ± 2.77	74.50 ± 3.83
After challenge				
Control	1.12 ± 0.02^a^	0.81 ± 0.08^a^	37.06 ± 2.66	91.26 ± 4.09
Sc	1.14 ± 0.01^ab^	1.12 ± 0.07^b^	33.71 ± 3.15	82.81 ± 4.50
Tb	1.16 ± 0.04^ab^	1.20 ± 0.09^bc^	31.46 ± 2.31	80.75 ± 3.97
Sc + Tb	1.21 ± 0.03^d^	1.21 ± 0.03^bc^	29.53 ± 2.20	84.66 ± 3.01
*P*-values				
Diet	<0.001	<0.001	0.002; control^(z)^, Sc^(yz)^, Tb^(xy)^, Sc + Tb^(x)^	0.001; control^(y)^, Sc^(x)^, Tb^(x)^, Sc + Tb^(x)^
Challenge	<0.001	<0.001	<0.001 ^*∗*^	<0.001 ^*∗*^
Interaction	0.027	0.027	0.879	0.455

Different superscripts “a,” “b,” “c,” and “d” within a column show significant differences among the treatments (Duncan). Asterisk shows significant elevation after the challenge (main effect of challenge). Different superscript, “x,” “y,” and “z” show significant difference among the dietary treatments (main effect of diet).

**Table 5:** Plasma immunological parameters of Nile tilapia fed with diets containing *S. cerevisiae* (Sc), costmary essential oil (Tb), and *S. cerevisiae* + costmary essential oil (Sc + Tb) and challenged with *A. hydrophila* (*n* = 3).

	Lysozyme (U/mL)	Total Ig (mg/mL)	ACH50 (U/mL)
Before challenge			
Control	23.96 ± 2.04	14.51 ± 1.05	124.14 ± 2.12^c^
Sc	26.87 ± 1.36	16.08 ± 0.35	124.61 ± 1.23^c^
Tb	29.17 ± 2.55	17.50 ± 0.24	129.23 ± 2.54^d^
Sc + Tb	27.78 ± 0.93	18.74 ± 0.38	131.02 ± 1.34^d^
After challenge			
Control	18.50 ± 2.24	13.25 ± 0.31	110.71 ± 0.94^a^
Sc	23.19 ± 1.68	16.17 ± 0.32	116.43 ± 1.56^b^
Tb	25.45 ± 2.72	17.38 ± 0.15	121.74 ± 0.83^c^
Sc + Tb	23.22 ± 1.36	17.62 ± 0.34	122.93 ± 1.53^c^
*P*-values			
Diet	0.001; control^(w)^, Sc^(x)^, Tb^(x)^, Sc + Tb^(x)^	<0.001; control^(w)^, Sc^(x)^, Tb^(y)^, Sc + Tb^(z)^	<0.001
Challenge	<0.001 ^*∗*^	0.487	<0.001
Interaction	0.841	0.054	0.019

Different superscripts “a,” “b,” “c,” and “d” within a column show significant differences among the treatments (Duncan).  ^*∗*^Shows significant elevation after the challenge (main effect of challenge). Different superscripts “w,” “x,” “y,” and “z” show significant difference among the dietary treatments (main effect of diet). Ig, immunoglobulin; ACH50, alternative complement activity.

**Table 6:** Plasma immunological parameters of Nile tilapia fed with diets containing *S. cerevisiae* (Sc), costmary essential oil (Tb), and *S. cerevisiae* + costmary essential oil (Sc + Tb) challenged with *A. hydrophila* (*n* = 3).

	MDA (nmol/mL)	GPx (U/mL)	SOD (U/mL)	CAT (U/mL)
Before challenge				
Control	184.53 ± 2.23	223.27 ± 1.89^b^	71.47 ± 1.28^a^	66.25 ± 2.72^c^
Sc	168.42 ± 3.28	228.48 ± 1.99^b^	78.70 ± 1.90^b^	65.29 ± 2.13^c^
Tb	166.72 ± 1.34	274.39 ± 2.45^e^	83.02 ± 2.91^bc^	56.17 ± 2.50^b^
Sc + Tb	168.26 ± 1.76	266.08 ± 2.60^d^	82.18 ± 1.88^bc^	52.88 ± 3.65^ab^
After challenge				
Control	192.97 ± 1.50	216.51 ± 3.39^a^	69.57 ± 4.11^a^	68.29 ± 2.65^c^
Sc	174.63 ± 2.96	242.10 ± 5.43^c^	84.26 ± 2.09^c^	57.17 ± 0.98^b^
Tb	176.10 ± 1.91	288.50 ± 2.90^f^	94.13 ± 1.55^d^	50.69 ± 2.15^a^
Sc + Tb	175.12 ± 1.76	295.79 ± 3.64^g^	98.96 ± 2.61^e^	49.52 ± 1.75^a^
*P*-values				
Diet	<0.001; control^(y)^, Sc^(x)^, Tb^(x)^, Sc + Tb^(x)^	<0.001	<0.001	<0.001
Challenge	<0.001 ^*∗*^	<0.001	<0.001	0.002
Interaction	0.593	<0.001	<0.001	0.015

Different superscripts “a,” “b,” “c,” “d,” “e,” “f,” and “g” within a column show significant differences among the treatments (Duncan). Asterisk shows significant elevation after the challenge (main effect of challenge). Different superscripts “x” and “y” show significant difference among the dietary treatments (main effect of diet). MDA, malondialdehyde; GPx, glutathione peroxidase; SOD, superoxide dismutase; and CAT, catalase.

## Data Availability

The data are available from the corresponding author upon reasonable request.
